# Watering CanMEDS flowers

**Published:** 2017-06-30

**Authors:** Sarah Voll, Taufik A. Valiante

**Affiliations:** 1 MD Program, Faculty of Medicine, University of Toronto, Ontario, Canada; 2 Division of Neurosurgery, Department of Surgery, University of Toronto, Ontario, Canada; 3 Krembil Research Institute, University Health Network, Toronto, Ontario, Canada

## Abstract

We expand on the discourse related to nature surrounding Canadian medical training and the CanMEDS flower. We advance the notion that a major contributor to burnout is the increasing complexity and segregation of not only the medical profession, but society as a whole, leading to choice overload and an overemphasis on complex, data driven, rational decision making. We then propose that the key to watering CanMEDS flowers and preventing physician burnout lies in getting back to the basics of human behaviour.

The CanMEDs framework, depicted as a flower, has initiated a discourse related to nature in medical training.^1^ Each year, hundreds of buds begin their medical school journey. The goal of medical education is to care for those buds so that they blossom into beautiful flowers, i.e., good doctors. At a time when burnout and mental illness are of utmost concern, how can we water CanMEDS flowers and prevent them from wilting?

Economists and psychologists have been saying for years that having too many choices may lead to less satisfaction with the decision made, particularly when people have a lack of familiarity with certain choice items.^2^ When an applicant applies to medical school they are applying to “medicine.” All specialties are lumped into one category and the applicant is selected based on traits and experiences that make them well suited to the profession as a whole. However, the amount of choices to be made regarding their future career may blur their original purpose for applying to medicine.

These choices are a reflection of an increasingly segmented society. Instead of deciding which book to read, we are deciding which webpage to look at. We buy songs instead of albums. And as we decide between smaller and smaller parts of a whole, we lose depth of understanding about the whole concept from which the part is extracted. We may be losing track of the big picture, and most importantly, our big picture, i.e., our sense of identity as a doctor and our satisfaction with life. Even the concept of being a doctor has been further broken down into seven CanMEDS roles. Could it be that even though we are all competent scholars, leaders, collaborators, communicators, professionals, health advocates, and medical experts we are losing track of what it means to be a doctor?

One way to prevent burnout may be to put some pieces back together and simplify. In his book Start with Why,^3^ Simon Sinek speaks of a golden circle with “why” at the centre, “how” in the middle and “what” on the outside. Sinek posits that although most people can tell you “what” they do and “how” they do it, very few people know “why” they do what they do. However, he makes a compelling argument that the “why” is critical for success, since people think and act from the inside out. We propose that the “why” is the central driving force that enables one to carry out the “what” with ease. It is the “why” that enables good-doctor-ness and prevents burnout. A doctor’s “why” is at the heart of their identity as a medical expert.

In an increasingly complex, segmented society, we need to take a step back and return to the basics of human behaviour, to the broad concept of the “doctor,” to meaning, character and our “why.” Somewhere deep in all of us is our driving force for professional fulfillment. We need to find it, nurture it, and never lose it.
